# Hemodynamic Variability in Aortic Stenosis and Regurgitation During Transcatheter Aortic Valve Replacement With Self-Expanding Valves

**DOI:** 10.1155/cdr/8821435

**Published:** 2025-10-23

**Authors:** Mingfei Li, Jianing Fan, Shasha Chen, Dawei Lin, Xiaochun Zhang, Wenzhi Pan, Daxin Zhou, Junbo Ge

**Affiliations:** ^1^Department of Cardiology, Zhongshan Hospital, Fudan University, Shanghai Institute of Cardiovascular Diseases, Shanghai, China; ^2^State Key Laboratory of Cardiovascular Diseases, Zhongshan Hospital, Fudan University, Shanghai, China; ^3^NHC Key Laboratory of Ischemic Heart Diseases, Shanghai, China; ^4^Key Laboratory of Viral Heart Diseases, Chinese Academy of Medical Sciences, Shanghai, China; ^5^National Clinical Research Center for Interventional Medicine, Shanghai, China

**Keywords:** aortic regurgitation, aortic stenosis, self-expanding valves, transcatheter aortic valve replacement

## Abstract

**Objective:**

This study investigated the impact of pathological differences between aortic stenosis (AS) and aortic regurgitation (AR) on hemodynamic outcomes following transcatheter aortic valve replacement (TAVR), with a focus on the performance of self-expanding valves relative to annular anatomy.

**Methods:**

In this single-center, retrospective study, patients undergoing TAVR for AS or AR were stratified by annulus area into smaller (< 430 cm^2^) and larger (≥ 430 cm^2^) subgroups. Valve sizing was based on annular dimensions (≥ 27 mm for AR/smaller annulus; < 27 mm for AS subgroups). Hemodynamic parameters (aortic valve area [AVA], pressure gradients, and velocity) and prosthesis characteristics (sheath size and compression ratio) were evaluated pre- and postoperatively, with 1-year follow-up.

**Results:**

The AR group required larger sheaths (*p* = 0.006) and demonstrated superior hemodynamics compared to the AS group: larger postoperative AVA (3.0 ± 0.4 vs. 2.2 ± 0.5  and 2.1 ± 0.6 cm^2^ in larger and smaller annuli, respectively, *p* < 0.001); lower maximum (9.7 ± 4.3 vs. 15.8 ± 9.2 and 18.8 ± 10.8 mmHg in larger and smaller annuli, respectively, *p* < 0.001) and mean gradients (7.8 ± 4.4 mmHg vs. others, *p* < 0.001); and reduced aortic velocity (1.60 ± 0.43 vs. others, *p* = 0.038). Smaller annuli exhibited higher prosthesis compression (0.88 ± 0.04 vs. 0.84 ± 0.04 in AR and 0.8 ± 0.06 in larger annulus, *p* < 0.001), with 20% (*n* = 8) developing elevated transvalvular gradients (> 20 mmHg) at follow-up.

**Conclusions:**

One-year outcomes revealed distinct hemodynamic profiles post-TAVR between AR and AS groups based on annular size. Patients with AR exhibited more favorable valve performance, supporting TAVR in younger, low-risk patients with AR who have suitable annular anatomy.

## 1. Introduction

Aortic stenosis (AS) is a potentially life-threatening cardiac condition primarily caused by degenerative calcification of the valve leaflets or congenital bicuspid valve abnormalities, leading to leaflet thickening and stiffening. The resulting hemodynamic alterations in AS are primarily caused by elevated transvalvular pressure gradients, reduced ejection fraction, and decreased cardiac output [1, 2]. With technological advancements, transcatheter aortic valve replacement (TAVR) has become a well-established treatment for AS [3, 4].

In contrast, aortic regurgitation (AR) typically results from structural abnormalities of the valve leaflets or pathological changes in the aortic root. Hemodynamic changes in AR include increased pulmonary arterial pressure, elevated ventricular preload, and progressive left ventricular dysfunction. However, ejection fraction may be preserved during the early stages of AR [5, 6]. Despite these challenges, TAVR has shown potential in treating patients with AR by alleviating these hemodynamic changes [7, 8].

The anatomical features of AR, such as larger annulus size and less calcification, may lead to different hemodynamic responses compared to AS following TAVR [9]. These differences may have significant implications on the overall effectiveness and outcomes of the procedure.

In this study, we conducted a comprehensive analysis of postoperative outcomes in patients who received self-expanding valves (SEVs), focusing on hemodynamic improvements across patients with AR or AS with varying annulus sizes. This study was aimed at providing a more nuanced understanding of the performance of SEVs in these patients.

## 2. Methods

### 2.1. Patient Selection

This single-center, retrospective study included patients with AS and AR who underwent TAVR between January 2019 and January 2023. All patients were rigorously screened to confirm eligibility for inclusion and representativeness of the study cohort. The exclusion criteria were as follows:

1. Prior implantation of balloon-expandable valves
2. History of cardiac surgery
3. Prior valve-in-valve procedures
4. Inability to provide computed tomography (CT) or echocardiographic data
5. Lack of follow-up data

The exclusion criteria were established to ensure the accuracy and reliability of the study findings, with a focus on a homogeneous population receiving SEVs. The patients with AR who were selected for TAVR had definitive surgical indications but were deemed ineligible for surgery due to contraindications or high surgical risk, with anatomical suitability as confirmed by the heart team.

### 2.2. Echocardiography and CT

All patients underwent preoperative transthoracic echocardiography (TTE) to assess aortic valve pathology. AS was diagnosed based on one or more of the following: effective orifice area < 1.0 cm^2^; mean pressure gradient ≥ 40 mmHg; peak velocity > 4.0 m/s; or dimensionless index ≤ 0.25. TTE often revealed leaflet thickening or calcification. AR was defined by the presence of retrograde blood flow through the aortic valve, with severe AR identified by a regurgitant fraction > 50%, regurgitant volume > 60 mL/beat, or effective regurgitant orifice area ≥ 0.30 cm^2^ [10, 11]. Typical echocardiographic features included left ventricular dilation and abnormal valve morphology.

For eligible patients, preoperative multidetector CT was performed to evaluate procedural feasibility and guide valve selection. Annular dimensions, including circumference, diameter, and area, were measured and used for prosthesis sizing. These measurements were essential for understanding the condition of the patient and planning the procedure.

### 2.3. TAVR Procedure

TAVR was performed via transfemoral access under fluoroscopic and transesophageal echocardiography (TEE) guidance. The delivery system was deployed to the aortic root, and the SEV prosthesis was radially expanded for optimal annular apposition. The delivery catheter was subsequently retracted under continuous hemodynamic monitoring. Hemostasis was achieved using a preclosed technique with ProGlide devices.

### 2.4. Classification

Patients with pure AR, as confirmed by echocardiography, were categorized into the AR group. Patients with AS were stratified by annular area: larger annulus (≥ 430 mm^2^) and smaller annulus (< 430 mm^2^) groups [12]. This classification facilitated subgroup comparisons.

### 2.5. Data Collection and Follow-Up

Collected preoperative data included demographics (gender and age), comorbidities, imaging findings (CT), laboratory results, and cardiac catheterization data. Intraoperative parameters (TEE findings and valve model) and postoperative follow-up results were also recorded. This comprehensive dataset supported detailed outcome assessment and tailored treatment planning.

### 2.6. Data Management and Statistical Analysis

Statistical analyses were performed using parametric tests (*t*-tests and analysis of variance) for normally distributed data and nonparametric tests (Wilcoxon rank-sum test, Mann–Whitney *U* test, or Kruskal–Wallis) for nonnormally distributed data. Continuous variables were expressed as means with standard deviations or medians with interquartile ranges. Categorical variables were analyzed using the chi-square test or Fisher's exact test, as appropriate.

## 3. Results

### 3.1. Study Population and Group Stratification

**Figure 1:**
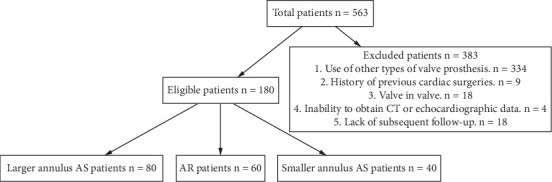
Patient selection flowchart. Abbreviations: AR: aortic regurgitation; AS: aortic stenosis; CT: computed tomography.

This retrospective study included 563 consecutive patients who underwent TAVR. After applying the exclusion criteria to 383 patients, 180 who underwent TAVR with VitaFlow SEVs (MicroPort Scientific Co. Ltd., Shanghai, China) were enrolled and stratified into three anatomical cohorts: AR group (*n* = 60, 33.3%), larger annulus group (*n* = 80, 44.4%), and smaller annulus group (*n* = 40, 22.2%), based on annular dimensions and valvular characteristics (Figure 1).

### 3.2. Baseline Demographic and Clinical Characteristics

As shown in Table 1, significant intergroup differences were observed. The AR group had significantly older patients (74.2 ± 9.6 vs. 78.3 ± 7.6 and 77.6 ± 7.1 years, *p* = 0.024) and lower body weight (56.3 ± 11.8 vs. 64.3 ± 11.4 and 58.8 ± 10.2 kg, *p* = 0.006) compared with the larger and smaller annuli groups. Height showed a trend toward significance (161.1 ± 9.6 vs. 165.3 ± 7.9 and 160.5 ± 8.6 cm, *p* = 0.056), while body mass index differed significantly (21.2 vs. 23.4 and 23.1 kg/m^2^, *p* = 0.041). Other significant differences included body surface area (*p* = 0.009), diabetes prevalence (*p* = 0.035), and sex distribution (*p* = 0.002). Although not statistically significant, coronary artery disease prevalence was higher in the larger annulus group (87.5%) than the AR (65.4%) and smaller annulus (75.0%) groups (*p* = 0.107). Other comorbidities were comparable across groups.

### 3.3. Baseline Echocardiographic Parameters

Table 2 shows key anatomical and functional differences across groups. Left ventricular end-diastolic dimension and aortic valve area (AVA) were largest in the AR group (57.6 ± 7.5 mm and 3.1 ± 0.3 cm^2^, respectively). Hemodynamic parameters showed lower gradients and velocity in the AR group (max/mean: 10.2 ± 4.7/4.6 ± 2.7 mmHg; velocity: 1.2 ± 0.2 m/s) compared to the larger and smaller annuli groups. Annular diameter was largest in the AR group (22.9 ± 2.1 mm). However, no significant differences were found in the left atrial diameter, pulmonary arterial systolic pressure (PASP), and left ventricular ejection fraction (LVEF).

### 3.4. CT Measurements and Intraoperative Data

As shown in Table 3, CT revealed significant anatomical differences in annular area and circumference between the AR and smaller annulus groups. Prosthesis diameter selection followed distinct patterns: the AR group exclusively received ≥ 27-mm devices, the smaller annulus group used only < 27 mm, and the larger annulus group used both sizes (*p* < 0.001). The annulus-to-prosthesis diameter ratio showed significant differences across groups (*p* < 0.001), with the AR group having the lowest compression ratio (0.80 ± 0.06 vs. 0.84 ± 0.04 and 0.88 ± 0.04 in the larger and smaller annuli groups). Preoperative left ventricular pressure was significantly lower in the AR group (122.1 ± 22.6 mmHg) than in the larger (176.7 ± 43.5 mmHg) and smaller annuli groups (177.9 ± 28.0 mmHg, *p* < 0.001). The AR group also required larger sheath sizes (20.1 ± 1.6 Fr vs. other groups, *p* = 0.006). While the pre- and postoperative systolic aortic pressures did not differ significantly, the overall hemodynamic profiles highlighted marked pathophysiological differences between groups.

### 3.5. Postoperative Hemodynamic Outcomes and In-Hospital Complications

Postoperative LVEF was significantly lower in the AR group (53.6% ± 13.9%) than in the larger (58.5% ± 8.4%) and smaller annuli groups (62.3% ± 10.5%, *p* < 0.001). The AR group also had the largest postoperative AVA (3.0 ± 0.4 cm^2^ vs. other groups, *p* < 0.001) and the lowest gradients compared to the larger and smaller annuli groups: maximum (9.7 ± 4.3 vs. 15.8 ± 9.2 and 18.8 ± 10.8 mmHg, *p* < 0.001) and mean gradients (4.3 ± 2.0 vs. 7.8 ± 4.4 and 9.2 ± 5.3 mmHg, *p* < 0.001). Postoperative aortic valve velocity was also lowest in the AR group (1.6 ± 0.4 m/s vs. other groups, *p* = 0.038). No severe valvular complications, such as valve thickening, thrombosis, or significant transvalvular pressure gradient elevation, were observed. In-hospital complications were most frequent in the larger annulus group (7.5%). New permanent pacemaker implantation rates were more common in the larger (5.0%) and smaller annuli (5.0%) groups than in the AR group (3.3%) (Table 4).

### 3.6. Outcomes at 1-Year Follow-Up

**Figure 2:**
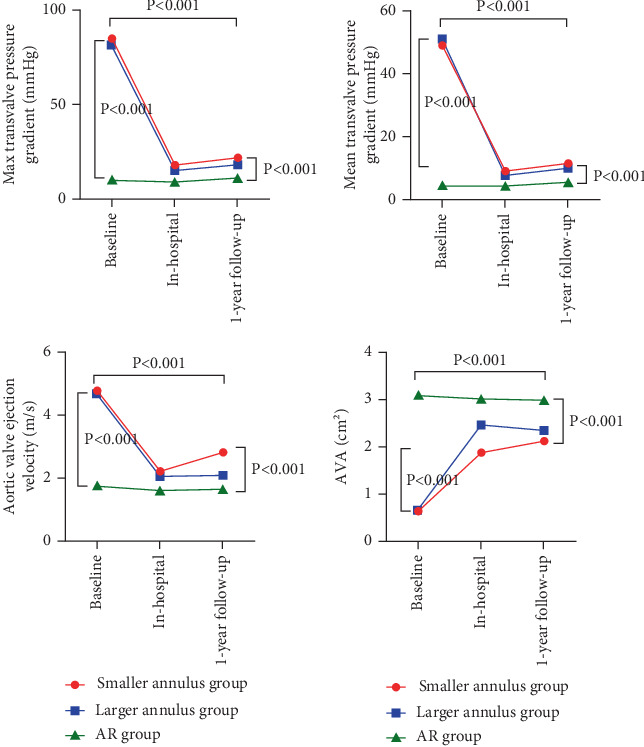
The valve function improvement in the AR group, the larger annulus group, and the smaller annulus group. (a) Comparison of the maximum aortic valve pressure gradient among the three groups. (b) Comparison of the mean aortic valve pressure gradient among the three groups. (c) Comparison of the peak aortic valve ejection flow velocity among the three groups. (d) Comparison of the aortic valve orifice area among the three groups. Abbreviations: AR: aortic regurgitation; AVA: aortic valve area.

At 1 year, intergroup differences in TTE parameters persisted (Table 5). LVEF remained lowest in the larger annulus group (56.7% ± 11.4%) compared to the smaller annulus (63.7% ± 8.5%) and AR (63.9% ± 8.4%) groups (*p* = 0.003). Valvular hemodynamics displayed distinct patterns: aortic valve velocity (1.6 ± 0.3 vs. 2.1 ± 0.5 vs. 2.8 ± 2.4 m/s, *p* = 0.004), peak gradient (18.6 ± 10.8 vs. 10.3 ± 4.8 vs. 22.7 ± 10.7 mmHg, *p* < 0.001), and mean gradient (5.7 ± 2.1 vs. 9.9 ± 6.1 vs. 11.7 ± 6.4 mmHg, *p* < 0.001) all differed significantly. AVA decreased progressively across the groups (3.0 ± 0.6 vs. 2.4 ± 0.6 vs. 2.1 ± 0.5 cm^2^, *p* < 0.001; Figure 2). PASP was significantly elevated in the smaller annulus group compared to the other groups (41.3 ± 10.7 vs. 35.3 ± 7.6 vs. 35.4 ± 7.7 mmHg, *p* = 0.006). Notably, 20% of the patients (*n* = 4) in the smaller annulus group developed an elevated mean gradient of > 20 mmHg, representing the only cohort with this complication. There were no significant differences in all-cause mortality among the three groups.

## 4. Discussion

This study yielded several clinically significant observations regarding TAVR outcomes. First, patients with AR exhibited superior early- and intermediate-term hemodynamic performance compared to those with AS, irrespective of annulus size. This advantage was demonstrated by lower transvalvular pressure gradients, reduced peak systolic flow velocities, and larger effective orifice areas in patients with AR than in those with small annulus AS. Second, both the large annulus AS and AR groups showed stable hemodynamic profiles, with no cases of significant postoperative transvalvular gradient elevation. In contrast, 20% (8/40) of patients in the small annulus AS group developed elevated gradients (> 20 mmHg) during follow-up, including one case of clinically significant leaflet thickening that required ongoing surveillance. Third, during the interim follow-up period, despite distinct anatomical and valvular characteristics, there were no significant intergroup differences in the incidence of key clinical events, suggesting favorable short- to midterm outcomes across all cohorts. These findings provide valuable insights into the hemodynamic characteristics and outcomes of TAVR in patients with varying aortic valve pathologies and annular anatomies.

As the TAVR patient population continues to age, the long-term functional integrity of bioprosthetic valves becomes a critical concern. Early valve dysfunction often signals the onset of premature aortic valve deterioration and increases the risk of reintervention [13–15]. Hemodynamic deterioration serves as a crucial indicator of prosthesis–annulus mismatch or valve thrombosis [16, 17]. This plays a pivotal role in the assessment of valve performance and longevity.

Postoperative hemodynamic performance is influenced by multiple factors, including annulus size and prosthesis selection. Patients with smaller annular sizes are particularly susceptible to hemodynamic deterioration, which can increase the risk of adverse outcomes. Over time, increased cardiac workload can impair ventricular function, ultimately affecting patient survival and quality of life [16–19]. Therefore, accurate preoperative imaging and tailored valve selection are crucial to minimizing the incidence of hemodynamic deterioration and improving patient outcomes.

Baseline comparisons revealed that patients in the larger annulus group were more likely to be male and have greater body weight and body surface area, which is consistent with previous studies [12, 20]. Patients with AR exhibited lower body mass index, suggesting more advanced cardiac cachexia and poorer cardiac function, and lower LVEF (54.5% ± 13.2% vs. 56.7% ± 11.8% vs. 52.8% ± 11.7%, *p* = 0.065), although the difference in LVEF was not statistically significant.

Echocardiographic and hemodynamic assessments showed that patients with AS and smaller annuli had the poorest baseline status, including smaller AVA, larger transvalvular pressure gradients, and higher peak aortic valve ejection flow velocities. These findings are consistent with previous studies that identified a smaller annulus as an independent predictor of prosthesis–patient mismatch, which is associated with an increased risk of valve thrombosis, structural deterioration, and mortality [12, 16].

Patients with AR exhibited better post-TAVR valve function. TAVR effectively reduces anteroposterior cardiac load and displaces the diseased native valve. However, limited research exists on TAVR outcomes in AR, especially considering the significant differences in annulus size and leaflet calcification compared with AS, warranting further investigation.

TAVR remains a valuable option for patients with AR who have high surgical risk or anatomically unsuitable conditions [21]. The preserved hemodynamic performance and structural valve integrity post-RAVR may reduce the risk of degeneration and reintervention. These findings support the use of TAVR in younger and lower-risk populations with AR who were previously not considered suitable for surgical treatment.

## 5. Limitations

Our study had several limitations. First, the number of participants was small, particularly for the AR group. The absence of multivariate adjustment may have introduced bias. Second, the lack of standardized multidimensional criteria for annular sizing may oversimplify anatomical influences on outcomes. Third, the limited follow-up duration (median of 24 months) precludes long-term prognostic analysis, including progression of valve deterioration. Fourth, only one valve type (VitaFlow) was used, which enhances consistency but limits generalizability beyond the local context. Lastly, the absence of postoperative imaging, such as CT for thrombus detection, may have led to underdiagnosis of subclinical dysfunction, potentially underestimating the incidence of hemodynamic deterioration. Further studies addressing these limitations should be done to validate our findings.

## 6. Conclusion

This study is the first to compare short- and midterm outcomes of TAVR with respect to valve function in patients with AR and AS across varying annular sizes. Our findings suggest that patients with AR experience more favorable postoperative hemodynamic improvement, independent of annular size. These results provide evidence supporting the use of TAVR in low-risk populations with AR, especially in younger age groups.

## Figures and Tables

**Table 1:** Patient demographics and baseline characteristics.

**Characteristic**	**Groups**			**p** **value**
	**Regurgitation group, ** **N** = 60	**Larger annulus group, ** **N** = 80	**Smaller annulus group, ** **N** = 40	
Age (years)	74.2 ± 9.6	78.3 ± 7.6	77.6 ± 7.1	0.024
Male	24 (40.0%)	56 (70.0%)	10 (25.0%)	0.002
Height (cm)	161.1 ± 10.8	165.3 ± 7.9	160.5 ± 8.6	0.056
Weight (kg)	56.3 ± 11.8	64.3 ± 11.4	58.8 ± 10.2	0.006
BMI (kg/m^2^)	21.2 ± 3.0	23.4 ± 3.9	23.1 ± 4.4	0.041
BSA (m^2^)	1.54 ± 0.19	1.67 ± 0.17	1.57 ± 0.14	0.009
Smoke	6 (10.0%)	6 (7.5%)	2 (5.0%)	0.891
Diabetes	8 (13.4%)	14 (17.5%)	14 (35.0%)	0.035
Hypertension	40 (66.7%)	40 (50.0%)	26 (65.0%)	0.307
AF	10 (16.7%)	18 (22.5%)	10 (25.0%)	0.799
CAD	34 (65.4%)	70 (87.5%)	30 (75.0%)	0.107
MI	0 (0.0%)	4 (5.0%)	0 (0.0%)	0.501
Stroke	0 (0.0%)	2 (2.5%)	2 (5.0%)	0.700
PAD	0 (0.0%)	4 (5.0%)	6 (15.0%)	0.088
COPD	2 (3.3%)	6 (7.5%)	2 (5.0%)	0.846
PCI	8 (13.3%)	8 (10.0%)	8 (20.0%)	0.568
CABG	2 (3.3%)	0 (0.0%)	0 (0.0%)	0.556
PPMI	2 (3.3%)	4 (5.0%)	0 (0.0%)	0.796
NYHAIII-IV	56 (93.4%)	70 (87.5%)	32 (80.0%)	0.493
LBBB	2 (3.3%)	0 (0.0%)	0 (0.0%)	0.556
RBBB	2 (3.3%)	6 (7.5%)	0 (0.0%)	0.548
Creatinine	99.4 ± 39.6	95.8 ± 30	90.9 ± 24.3	0.587
Nt-ProBNP (pg/mL)	3308 ± 5660	5322 ± 6478	2667 ± 3731	0.173
Hemoglobin (g/dL)	121.7 ± 17.6	124.5 ± 20.2	121.2 ± 15.6	0.728
TnT (ng/mL)	0.030 ± 0.038	0.039 ± 0.035	0.024 ± 0.013	0.228

Abbreviations: AF, atrial fibrillation; BMI, body mass index; BSA, body surface area; CABG, coronary artery bypass grafting; CAD, coronary artery disease; COPD, chronic obstructive pulmonary disease; LBBB, left bundle branch block; MI, myocardial infarction; NYHAIII-IV, New York Heart Association Functional Classification III-IV; PADs, peripheral vascular diseases; PCI, percutaneous coronary intervention; PPMI, permanent pacemaker implantation; RBBB, right bundle branch block; TnT, troponin T.

**Table 2:** Baseline echocardiographic parameters.

**Characteristic**	**Groups**			**p** **value**
	**Regurgitation group, ** **N** = 60	**Larger annulus group, ** **N** = 80	**Smaller annulus group, ** **N** = 40	
LAD (mm)	43.7 ± 6.6	46.8 ± 7.2	46.9 ± 12.8	0.241
LVEDD (mm)	57.6 ± 7.5	52.8 ± 7.1	46.3 ± 8.5	< 0.001
AVA (cm^2^)	3.1 ± 0.3	0.67 ± 0.18	0.65 ± 0.16	< 0.001
PASP (mmHg)	40 ± 10.6	45.3 ± 14.6	47.7 ± 17.3	0.107
Maximum aortic valve systolic pressure gradient (mmHg)	10.2 ± 4.7	82.9 ± 26.4	85.8 ± 36.6	< 0.001
Mean aortic valve systolic pressure gradient (mmHg)	4.6 ± 2.7	51.6 ± 18.4	49.4 ± 22.7	< 0.001
Aortic valve ejection velocity (m/s)	1.2 ± 0.2	4.7 ± 0.7	4.8 ± 0.8	< 0.001
Annulus diameter (mm)	22.9 ± 2.1	22.3 ± 1.8	20.8 ± 1	< 0.001
Calcification	0 (0%)	80 (100.0%)	40 (100.0%)	< 0.001
LVEF (%)	54.5 ± 13.2	56.7 ± 11.8	62.8 ± 11.7	0.065

Abbreviations: AVA, aortic valve area; LAD, left atrial diameter; LVEDD, left ventricular end-diastolic diameter; LVEF, left ventricular ejection fraction; PASP, pulmonary artery systolic pressure.

**Table 3:** CT measurements and intraoperative information.

**Characteristic**	**Groups**			**p** **value**
	**Regurgitation group, ** **N** = 60	**Larger annulus group, ** **N** = 80	**Smaller annulus group, ** **N** = 40	
CT measurements				
Aortic annulus area (mm^2^)	513.3 ± 73.7	511.8 ± 69.2	368.8 ± 51.7	< 0.001
Circumference of valve annulus (mm)	80.8 ± 6.2	84.4 ± 6.7	72.6 ± 6.4	< 0.001
Prosthetic valve size				< 0.001
21	0 (0.0%)	6 (7.5%)	4 (10.0%)	
24	0 (0.0%)	32 (40.0%)	36 (90.0%)	
27	30 (50.0%)	32 (40.0%)	0 (0.0%)	
30	30 (50.0%)	10 (12.5%)	0 (0.0%)	
Ratio of annulus to prosthesis diameter	0.80 ± 0.06	0.84 ± 0.04	0.88 ± 0.04	0.001
Preoperative aortic systolic pressure	117.6 ± 25.6	114.2 ± 27.5	112 ± 21.4	0.741
Preoperative left ventricular pressure	122.1 ± 22.6	176.7 ± 43.5	177.9 ± 28	0.001
Postoperative aortic systolic pressure	124.5 ± 20.1	123.7 ± 23.3	123.7 ± 20.4	0.984
Sheath size	20.1 ± 1.6	18.5 ± 2.1	18.9 ± 2.5	0.006
Preoperative expansion	0 (0.0%)	78 (97.5%)	38 (95.0%)	0.001
Postoperative expansion	0 (0.0%)	30 (37.5%)	10 (25.0%)	0.008

**Table 4:** Postoperative in-hospital examination and adverse event.

**Characteristic**	**Groups**			**p** **value**
	**Regurgitation group, ** **N** = 60	**Larger annulus group, ** **N** = 80	**Smaller annulus group, ** **N** = 40	
TTE				
Valve thickening	0 (0.0%)	0 (0.0%)	0 (0.0%)	> 0.999
Valve thrombosis	0 (0.0%)	0 (0.0%)	0 (0.0%)	> 0.999
LVEF (%)	53.6 ± 13.9	58.5 ± 8.4	62.3 ± 10.5	< 0.006
PASP (mmHg)	35.3 ± 7.7	38 ± 9.7	36.2 ± 7.9	0.321
Aortic valve ejection velocity (m/s)	1.6 ± 0.4	2.1 ± 0.6	2.2 ± 0.6	0.038
AVA (cm^2^)	3.0 ± 0.4	2.5 ± 0.6	1.9 ± 0.3	< 0.001
Max transvalve pressure gradient (mmHg)	9.7 ± 4.3	15.8 ± 9.2	18.8 ± 10.8	< 0.001
Mean transvalve pressure gradient (mmHg)	4.3 ± 2	7.8 ± 4.4	9.2 ± 5.3	< 0.001
Complications				
Total complications	3 (3.3%)	6 (7.5%)	2 (5.0%)	0.642
Mortality	0 (0.0%)	0 (0.0%)	0 (0.0%)	> 0.999
Bleeding	0 (0.0%)	2 (2.5%)	0 (0.0%)	> 0.999
Acute kidney injury	0 (0%)	0 (0.0%)	0 (0.0%)	> 0.999
New-onset PPMI	2 (3.3%)	4 (5.0%)	2 (5.0%)	0.349
Stroke	0 (0.0%)	0 (0.0%)	0 (0.0%)	> 0.999
Myocardial infarction	0 (0.0%)	0 (0.0%)	0 (0.0%)	> 0.999
New-onset AF	0 (0.0%)	0 (0.0%)	0 (0.0%)	> 0.999
Peripheral vascular complications	0 (0.0%)	0 (0.0%)	0 (0.0%)	> 0.999
Coronary occlusion	0 (0.0%)	0 (0.0%)	0 (0.0%)	> 0.999
Tearing of annulus	0 (0.0%)	0 (0.0%)	0 (0.0%)	> 0.999
Septicemia	0 (0.0%)	0 (0.0%)	0 (0.0%)	> 0.999

Abbreviations: AF, atrial fibrillation; AVA, aortic valve area; LVEF, left ventricular ejection fraction; PASP, pulmonary artery systolic pressure; PPMI, permanent pacemaker implantation; TTE, transthoracic echocardiogram.

**Table 5:** Outcomes at 1-year follow-up.

**Characteristic**	**Groups**			**p** **value**
	**Regurgitation group, ** **N** = 60	**Larger annulus group, ** **N** = 80	**Smaller annulus group, ** **N** = 40	
TTE				
Valve thickening	0 (0.0%)	2 (2.6%)	0 (0.0%)	> 0.999
Valve thrombosis	0 (0.0%)	0 (0.0%)	0 (0.0%)	> 0.999
LVEF (%)	56.7 ± 11.4	63.7 ± 8.5	63.9 ± 8.4	0.003
PASP (mmHg)	35.3 ± 7.6	35.4 ± 7.7	41.3 ± 10.7	0.006
Aortic valve ejection velocity (m/s)	1.6 ± 0.3	2.1 ± 0.5	2.8 ± 2.4	0.004
Max transvalve pressure gradient (mmHg)	10.3 ± 4.8	18.6 ± 10.8	22.7 ± 10.7	< 0.001
Mean transvalve pressure gradient (mmHg)	5.3 ± 2.1	9.9 ± 6.1	11.7 ± 6.4	< 0.001
AVA (cm^2^)	3 ± 0.6	2.4 ± 0.6	2.1 ± 0.5	< 0.001
All-cause mortality	2 (3.3%)	4 (5.0%)	2(5.0%)	0.783
Cardiogenic mortality	2 (3.3%)	4 (5.0%)	2 (5.0%)	> 0.9993
Bleeding	0 (0%)	0 (0.0%)	0 (0%)	> 0.999
Acute kidney injury	0 (0.0%)	0 (0.0%)	0 (0.0%)	> 0.999
Stroke	0 (0%)	0 (0%)	0 (0%)	> 0.999
Myocardial infarction	0 (0.0%)	0 (0%)	0 (0%)	> 0.999
New-onset AF	0 (0.0%)	0 (0%)	0 (0%)	> 0.999

Abbreviations: AF, atrial fibrillation; AVA, aortic valve area; LVEF, left ventricular ejection fraction; PASP, pulmonary artery systolic pressure; TTE, transthoracic echocardiogram.

## Data Availability

The data that support the findings of this study are not publicly available due to their containing information that could compromise the privacy of research participants but are available from the corresponding authors (email: daxin_zhou@163.com or email: pan.wenzhi@zs-hospital.sh.cn) upon reasonable request.

## References

[1] Lindman B. R., Clavel M.-A., Mathieu P. (2016). Calcific Aortic Stenosis. *Nature Reviews Disease Primers*.

[2] Blaser M. C., Kraler S., Lüscher T. F., Aikawa E. (2021). Multi-Omics Approaches to Define Calcific Aortic Valve Disease Pathogenesis. *Circulation Research*.

[3] Mack M. J., Leon M. B., Thourani V. H. (2019). Transcatheter Aortic-Valve Replacement With a Balloon-Expandable Valve in Low-Risk Patients. *New England Journal of Medicine*.

[4] Popma J. J., Deeb G. M., Yakubov S. J. (2019). Transcatheter Aortic-Valve Replacement With a Self-Expanding Valve in Low-Risk Patients. *New England Journal of Medicine*.

[5] Baumbach A., Patel K. P., Rudolph T. K., Delgado V., Treede H., Tamm A. (2024). Aortic Regurgitation: From Mechanisms to Management. *EuroIntervention*.

[6] Singh J. P., Evans J. C., Levy D. (1999). Prevalence and Clinical Determinants of Mitral, Tricuspid, and Aortic Regurgitation (the Framingham Heart Study). *American Journal of Cardiology*.

[7] Franzone A., Piccolo R., Siontis G. C. M. (2016). Transcatheter Aortic Valve Replacement for the Treatment of Pure Native Aortic Valve Regurgitation: A Systematic Review. *JACC: Cardiovascular Interventions*.

[8] Yoon S.-H., Schmidt T., Bleiziffer S. (2017). Transcatheter Aortic Valve Replacement in Pure Native Aortic Valve Regurgitation. *Journal of the American College of Cardiology*.

[9] Garcia S., Ye J., Webb J. (2023). Transcatheter Treatment of Native Aortic Valve Regurgitation: The North American Experience With a Novel Device. *JACC: Cardiovascular Interventions*.

[10] Grayburn P. A., Smith M. D., Harrison M. R., Gurley J. C., DeMaria A. N. (1988). Pivotal Role of Aortic Valve Area Calculation by the Continuity Equation for Doppler Assessment of Aortic Stenosis in Patients With Combined Aortic Stenosis and Regurgitation. *American Journal of Cardiology*.

[11] Waller B. F., Howard J., Fess S. (1994). Pathology of Aortic Valve Stenosis and Pure Aortic Regurgitation a Clinical Morphologic Assessment—Part I. *Clinical Cardiology*.

[12] Herrmann H. C., Mehran R., Blackman D. J. (2024). Self-Expanding or Balloon-Expandable TAVR in Patients With a Small Aortic Annulus. *New England Journal of Medicine*.

[13] Del Trigo M., Muñoz-Garcia A. J., Wijeysundera H. C. (2016). Incidence, Timing, and Predictors of Valve Hemodynamic Deterioration After Transcatheter Aortic Valve Replacement: Multicenter Registry. *Journal of the American College of Cardiology*.

[14] Alaour B., Tomii D., Nakase M. (2025). Hemodynamic Valve Deterioration After Transcatheter Aortic Valve Replacement: Incidence, Predictors, and Clinical Outcomes. *JACC: Cardiovascular Interventions*.

[15] Wang Y., Yu H., Shi Q., Xu M., Gao W. (2024). Spatiotemporal Analysis of the Effects of Exercise on the Hemodynamics of the Aorta in Hypertensive Rats Using Fluid-Structure Interaction Simulation. *Journal of Translational Internal Medicine*.

[16] Levesque T., Eltchaninoff H., Chabannes R. (2024). Impact of Prosthesis-Patient Mismatch After Transcatheter Aortic Valve Replacement. *Canadian Journal of Cardiology*.

[17] Bruno F., Rampone J. M., Islas F. (2024). Echocardiographic and Clinical Features of Patients Developing Prosthesis-Patient Mismatch After Transcatheter Aortic Valve Replacement: Insights From the Recovery-TAVR Registry. *American Heart Journal*.

[18] Del Trigo M., Muñoz-García A. J., Latib A. (2018). Impact of Anticoagulation Therapy on Valve Haemodynamic Deterioration Following Transcatheter Aortic Valve Replacement. *Heart*.

[19] Johnston D. R., Mehta C., Malaisrie S. C. (2024). Implanted Size and Structural Valve Deterioration in the Edwards Magna Bioprosthesis. *Annals of Cardiothoracic Surgery*.

[20] Eng M. H., Khalili H., Vavalle J. (2024). 3-Year Outcomes of Balloon-Expandable Valves: 20-mm vs Larger Valves (≥23 mm). *JACC: Cardiovascular Interventions*.

[21] Noble S., Mauler-Wittwer S. (2024). TAVR as an Alternative to SAVR for Pure Native Aortic Regurgitation. *Canadian Journal of Cardiology*.

